# Aerobic endurance training versus relaxation training in patients with migraine (ARMIG): study protocol for a randomized controlled trial

**DOI:** 10.1186/1745-6215-13-46

**Published:** 2012-04-27

**Authors:** Andreas Totzeck, Susanne Unverzagt, Maja Bak, Pierre Augst, Hans-Christoph Diener, Charly Gaul

**Affiliations:** 1 Headache Center, Department of Neurology, University Hospital Essen, University Duisburg-Essen, Hufelandstraße 55, 45147 Essen, Germany; 2 Institute of Medical Epidemiology, Biostatistics and Informatics, Martin-Luther-University Halle-Wittenberg, Magdeburger Straße 8, 06097, Halle/Saale, Germany; 3 Tvg Holsterhausen, Essen, Keplerstraße 93, 45147, Essen, Germany

## Abstract

**Background:**

Migraine is one of the most frequent headache diseases and impairs patients’ quality of life. Up to now, many randomized studies reported efficacy of prophylactic therapy with medications such as beta-blockers or anti-epileptic drugs. Non-medical treatment, like aerobic endurance training, is considered to be an encouraging alternative in migraine prophylaxis. However, there is still a lack of prospective, high-quality randomized trials. We therefore designed a randomized controlled trial to evaluate the efficacy of aerobic endurance training versus relaxation training in patients with migraine (ARMIG).

**Methods:**

This is a single-center, open-label, prospective, randomized trial. Sixty participants with migraine are randomly allocated to either endurance training or a relaxation group. After baseline headache diary documentation over at least 4 weeks, participants in the exercise group will start moderate aerobic endurance training under a sport therapist’s supervision at least 3 times a week over a 12-week period. The second group will perform Jacobson’s progressive muscle relaxation training guided by a trained relaxation therapist, also at least 3 times a week over a 12-week period. Both study arms will train in groups of up to 10 participants. More frequent individual training is possible. The follow-up period will be 12 weeks after the training period. The general state of health, possible state of anxiety or depression, impairments due to the headache disorder, pain-related disabilities, the headache-specific locus of control, and the motor fitness status are measured with standardized questionnaires.

**Discussion:**

The study design is adequate to generate meaningful results. The trial will be helpful in gaining important data on exercise training for non-medical migraine prophylaxis.

**Trial registration:**

The trial is registered at ClinicalTrials.gov: NCT01407861.

## Background

Migraine is the second most frequent primary headache. With over 10% of people in the German population affected, migraine is a widespread disease leading to high healthcare costs and has a major impact on patients’ quality of life. Prophylactic medication, such as beta-blockers, topiramate, or amitriptyline, is recommended for patients suffering from three or more headache attacks a month [1]. However, medical prophylaxis is not well accepted by many migraine patients due to experienced or anticipated adverse events. Many patients seek non-medical treatment for migraine [2,3]. Endurance training is recommended [4], in particular as part of multidisciplinary treatment programs for migraine treatment [5-7]. Until now there has been insufficient evidence of the efficacy of endurance training due to the lack of randomized trials. Several studies reported a reduction of migraine frequency and intensity with aerobic endurance training. A major shortcoming of these studies is small patient numbers and therefore low statistical power. Aerobic endurance training led to an increased pain threshold in experimentally-induced pain. Most of these trials were conducted during the 1980s. Measurement of plasma beta endorphin levels in only a small number of migraine patients during exercise training cannot replace randomized trials of efficacy [8].

Recently, a Swedish single-center migraine trial comparing exercise training to relaxation training and to medical treatment with topiramate reported data on the efficacy of exercise training for prophylactic migraine treatment [9]. However, randomized trials using different comparators, especially non-pharmacological treatments, are not yet available [10].

We therefore initiated a German single-center trial to study the prophylactic effect of moderate aerobic endurance training versus relaxation training in patients with migraine.

Relaxation training, such as Jacobson’s progressive muscle relaxation, has been investigated in several studies and is effective for migraine prophylaxis [3,11-13].

## Design

This is a single-center, open-label, prospective, randomized study on the prophylactic effect of moderate aerobic endurance training versus relaxation training in patients with migraine.

The procedures and design of the study were approved by the Medical Ethics Committee of the University of Duisburg-Essen in Essen, Germany (Approval number 11–4677) and the study is internationally registered (ClinicalTrials.gov, Registration number NCT01407861).

The study started in July 2011, and participants are recruited via local newspapers, study brochures or local television. Interested participants are informed about the study protocol. After written informed consent, a sport therapist will make an appointment to conduct an endurance test to determine the optimal training heart rate. Participants will be examined by a neurologist to confirm the diagnosis of migraine and to check inclusion and exclusion criteria (see below). Diagnosis of migraine is based on the International Classification of Headache Disorders criteria (ICHD-II) [14].

### Inclusion criteria

Inclusion criteria are as follows:

1. The patient’s signature is on the informed consent document (patients should each be given ample time to read the consent form, ask any questions they may have regarding the trial, and have a clear understanding of the trial and the procedures involved prior to signing the consent form)

2. Patients have a clinical diagnosis of migraine with or without aura according to ICHD-II criteria [14] at least 1 year prior to enrollment

3. Patients have a clinical diagnosis of migraine with or without aura and, in addition, a clinical diagnosis of tension-type headache (TTH) according to ICHD-II [14], if patients are able to differentiate between the two headache diagnoses and if migraine is more frequent in patients with TTH

4. Patients report at least three migraine days per month

### Exclusion criteria

Exclusion criteria are as follows:

1. Diseases or disabilities that disqualify for performing either aerobic endurance training or relaxation training (that is, severe asthma, pacemaker, major depression, specific physical handicap)

2. Language disabilities that disenable filling out questionnaires

3. Fewer than three migraine days per month

4. Pregnancy

5. Additional diagnosis of secondary headache according to the ICHD-II (that is, medication overuse headache)

6. Prophylactic headache medication started within the last 12 weeks

7. Regular performance of endurance training and/or relaxation training at least twice a week

Migraine patients who are stable on prophylactic medication and meet the inclusion and exclusion criteria may be enrolled. Participants will be asked to keep a headache diary during the whole study period. Randomization will be performed at the end of the 4-week baseline period. Each participant will receive a sealed envelope. Randomization sequence is composed by an independent statistician using computer-generated random numbers. Participants are randomized to either the endurance training or the relaxation training group (1:1). Overall, 60 participants are planned to be enrolled in the trial. This is considered as a pilot trial to prove the feasibility of such a trial design and to achieve data on efficacy of both therapies.

**Figure 1:**
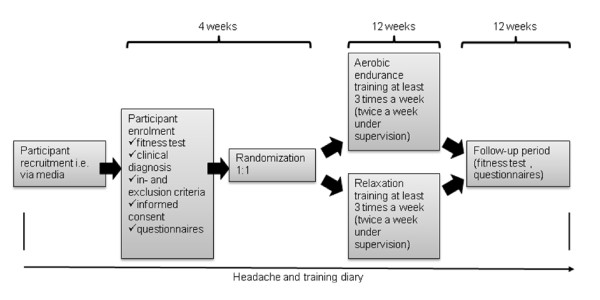
Flow chart of study protocol.

The treatment (training) period will begin after baseline headache diary documentation for at least 4 weeks to obtain baseline number of headache days. Training will be in groups of up to 10 participants (Figure 1).

### Fitness test

Each participant will perform a Physical Working Capacity Test (PWC) to test the aerobic performance capacity [15,16]. The test will be done using a computer-controlled ergometer with specialized software *Vitality System 6.0* (ERGO-FIT, Pirmasens, Germany). The results, defined as the mathematically-calculated performance at the threshold heart rate, are related to the participant’s body weight and thus converted to an individual value to receive optimal aerobic training heart rate. All test data will be printed out and filed in the participant’s case report form (CRF).

### Intervention: aerobic endurance training

Participants who are enrolled in the exercise group will perform endurance training twice a week for 12 weeks under the supervision of a trained sport therapist. The third training day during the week can be chosen individually. More frequent endurance training is possible. Each training session should be documented in the participant’s training headache and training diary. Endurance training consists of indoor cycling with a warm-up period of 10 min, a 30-min exercise period and a 5-min cool-down phase. Heart rate will be measured during exercise training to acquire optimal aerobic training conditions.

### Active comparison: relaxation training

Participants of the relaxation group (up to 10 people) will receive expert guidance in relaxation training twice a week over 12 weeks. A third training day during the week may also be chosen individually. More frequent relaxation training is again possible and should be documented in the participant’s training headache and training diary. Relaxation training is based upon Jacobson’s Progressive Muscle Relaxation (or PMR), an approved technique for reducing stress and anxiety by alternately tensing and relaxing groups of muscles. Participants will learn a short (about 10 min) and long (about 30 min) version of relaxation training. An audio compact disc containing the relaxation program will be provided to practice relaxation treatment at home.

Consistent participation in either exercise or relaxation is important and will be checked regularly. Up to two absent days in 4 weeks are tolerable.

### Headache and training diary

The headache diary should be kept every day during the trial to collect data about pain intensity, duration, accompanying symptoms, intake of analgesics or triptans, absent days from work/school, and training days for endurance training respectively relaxation training.

### Questionnaires

At baseline, after the 12-week training and 12 weeks after finishing the training period (follow-up), participants will be asked to fill out the following internationally-recognized, standardized questionnaires: the SF-12 (short-form 12), a questionnaire about the general state of health [17]; the HADS-D, a German version of the Hospital Anxiety and Depression Scale to capture possible states of anxiety or depression [18]; the HIT-6 (Headache Impact Test) to measure impairments due to headache disorders [19,20]; the Pain Disability Index (PDI) to measure pain-related disabilities [21]; the HSLC-D, a German version of the Headache-Specific Locus of Control Scale to gather information on the locus of control in headache patients [22]; the FFB-Mot, a self-survey on the motor fitness status consisting of 28 self-report items assessing four basic motor abilities, explicitly cardio-respiratory fitness, strength, flexibility, and coordination [23].

### Primary outcome measure

The primary outcome measure is a reduction of headache days in the last 4 weeks of the 12-week training compared to baseline (4 weeks before the start of training).

### Secondary outcome measures

Secondary outcome measures are as follows: number of headache days 12 weeks after finishing the training (follow-up); consumption of analgesics or triptans; increase in aerobic capability; impact on burden of disease; and the psychological impact of training.

### Statistical analysis

The aim of this pilot trial is to achieve data on the efficacy of both therapies. The effect of treatment on primary and secondary outcomes will be described by mean, standard deviations and confidence intervals per treatment group. These parameters and a definition of a clinically non-relevant difference will be used for sample size calculation of a randomized phase III trial to show non-inferiority of moderate aerobic endurance training versus relaxation training in patients with migraine [24].

## Discussion

This is a German single-center trial to study the prophylactic effect of moderate aerobic endurance training versus relaxation training in patients with migraine. We chose two active arms for comparison instead of inactive ‘waiting lists’ as placebo because inactive control groups would bear a high risk of withdrawal from the trial. Furthermore, relaxation training has proven to be effective in migraine treatment [3,11-13] and facilitates comparability, in contrast to medical treatment or ‘just taking a pill’, especially when done in a similar setting to exercise training. The study outcome depends on regular participation in either the exercise or the relaxation training group over a period of 12 weeks. Considering that all of the participants have not performed sports or relaxation training on regular basis before, a relevant drop-out rate cannot be ruled out. In the Swedish trial by Varkey *et al.*, only 85% completed the training, data on about 70% of the participants were collected at follow-up after 3 months, and only 50% of the participants returned questionnaires after 6 months [9]. Compared to the Swedish trial where relaxation training was performed individually, in our trial both exercise and relaxation training will mainly take place in a supervised group to rule out bias due to group dynamics. The overall number of 60 participants (30 in each study arm) may influence the significance of the results. However, Varkey *et al.* used an equal number of participants in their migraine exercise trial. Depending on our results, a multicenter trial with the same protocol may be planned.

## Trial status

Recruitment of participants started in July 2011 and is expected to end in summer 2012.

## Abbreviations

AT: Andreas Totzeck; CG: Charly Gaul; CRF: Case report form; FFB-Mot: Fragebogen zum motorischen Funktionsstatus (motor fitness questionnaire); HADS: Hospital Anxiety and Depression Scale; HIT-6: Headache Impact Test 6; HSLC: Headache-Specific Locus of Control Scale; ICHD-II: International Classification of Headache Disorders (second edition); PDI: Pain Disability Index; PMR: Jacobson’s Progressive Muscle Relaxation; PWC: Physical Working Capacity; SF-12: Short form 12.

## Competing interests

The authors declare that they have no competing interests regarding the study or this manuscript.

## Authors’ contributions

AT and CG contributed to the design and development of the trial protocol and were mainly responsible for writing the manuscript. All authors give substantial input to the manuscript draft; all of them read and approved the final version of the manuscript.

## References

[B1] Evers S Afra J Frese A Goadsby PJ Linde M May A Sandor PS EFNS guideline on the drug treatment of migraine–revised report of an EFNS task force Eur J Neurol 2009 16 968 981 10.1111/j.1468-1331.2009.02748.x 19708964

[B2] Gaul C Eismann R Schmidt T May A Leinisch E Wieser T Evers S Henkel K Franz G Zierz S Use of complementary and alternative medicine in patients suffering from primary headache disorders Cephalalgia 2009 29 1069 1078 10.1111/j.1468-2982.2009.01841.x 19366356

[B3] Holroyd KA Penzien DB Pharmacological versus non-pharmacological prophylaxis of recurrent migraine headache: a meta-analytic review of clinical trials Pain 1990 42 1 13 10.1016/0304-3959(90)91085-W 2146583

[B4] Busch V Gaul C Exercise in migraine therapy - is there any evidence for efficacy? A critical review Headache 2008 48 890 899 10.1111/j.1526-4610.2007.01045.x 18572431

[B5] Diener HC Gaul C Jensen R Gobel H Heinze A Silberstein SD Integrated headache care Cephalalgia 2011 31 1039 1047 10.1177/0333102411409075 21636624

[B6] Gaul C Visscher CM Bhola R Sorbi MJ Galli F Rasmussen AV Jensen R Team players against headache: multidisciplinary treatment of primary headaches and medication overuse headache J Headache Pain 2011 12 511 519 10.1007/s10194-011-0364-y 21779789 PMC3173636

[B7] Gaul C van Doorn C Webering N Dlugaj M Katsarava Z Diener HC Fritsche G Clinical outcome of a headache-specific multidisciplinary treatment program and adherence to treatment recommendations in a tertiary headache center: an observational study J Headache Pain 2011 12 475 483 10.1007/s10194-011-0348-y 21544647 PMC3139052

[B8] Koseoglu E Akboyraz A Soyuer A Ersoy AO Aerobic exercise and plasma beta endorphin levels in patients with migrainous headache without aura Cephalalgia 2003 23 972 976 10.1046/j.1468-2982.2003.00624.x 14984230

[B9] Varkey E Cider A Carlsson J Linde M Exercise as migraine prophylaxis: A randomized study using relaxation and topiramate as controls Cephalalgia 2011 31 1428 1438 10.1177/0333102411419681 21890526 PMC3236524

[B10] Darabaneanu S Overath CH Rubin D Luthje S Sye W Niederberger U Gerber WD Weisser B Aerobic exercise as a therapy option for migraine: a pilot study Int J Sports Med 2011 32 455 460 10.1055/s-0030-1269928 21472632

[B11] Niederberger U Kropp P Non pharmacological treatment of migraine Schmerz 2004 18 415 420 15300473 10.1007/s00482-004-0358-7

[B12] Penzien DB Rains JC Andrasik F Behavioral management of recurrent headache: three decades of experience and empiricism Appl Psychophysiol Biofeedback 2002 27 163 181 10.1023/A:1016247811416 12206049

[B13] Rains JC Penzien DB McCrory DC Gray RN Behavioral headache treatment: history, review of the empirical literature, and methodological critique Headache 2005 Suppl 2 S92 S109 15921506 10.1111/j.1526-4610.2005.4502003.x

[B14] The International Classification of Headache Disorders: 2nd edition Cephalalgia 2004 Suppl 1 9 160 14979299 10.1111/j.1468-2982.2003.00824.x

[B15] Perry SR Housh TJ Johnson GO Ebersole KT Bull AJ Heart rate and ratings of perceived exertion at the physical working capacity at the heart rate threshold J Strength Cond Res 2001 15 225 229 11710408

[B16] Wagner LL Housh TJ A proposed test for determining physical working capacity at the heart rate threshold Res Q Exerc Sport 1993 64 361 364 8235060 10.1080/02701367.1993.10608823

[B17] Ware J Kosinski M Keller SD A 12-Item short-form health survey: construction of scales and preliminary tests of reliability and validity Med Care 1996 34 220 233 10.1097/00005650-199603000-00003 8628042

[B18] Zigmond AS Snaith RP The hospital anxiety and depression scale Acta Psychiatr Scand 1983 67 361 370 10.1111/j.1600-0447.1983.tb09716.x 6880820

[B19] Bayliss MS Dewey JE Dunlap I Batenhorst AS Cady R Diamond ML Sheftell F A study of the feasibility of Internet administration of a computerized health survey: the headache impact test (HIT) Qual Life Res 2003 12 953 961 10.1023/A:1026167214355 14651414

[B20] Kosinski M Bayliss MS Bjorner JB Ware JE Garber WH Batenhorst A Cady R Dahlof CG Dowson A Tepper S A six-item short-form survey for measuring headache impact: the HIT-6 Qual Life Res 2003 12 963 974 10.1023/A:1026119331193 14651415

[B21] Tait RC Pollard CA Margolis RB Duckro PN Krause SJ The Pain Disability Index: psychometric and validity data Arch Phys Med Rehabil 1987 68 438 441 3606368

[B22] Martin NJ Holroyd KA Penzien DB The headache-specific locus of control scale: adaptation to recurrent headaches Headache 1990 30 729 734 10.1111/j.1526-4610.1990.hed3011729.x 2074167

[B23] Boes K Abel T Woll A Niemann S Tittlbach S Schott N Der Fragebogen zur Erfassung des motorischen Funktionsstatus (FFB-Mot) Diagnostica 2002 48 101 111 10.1026//0012-1924.48.2.101

[B24] Wellek S Testing Statistical Hypotheses of Equivalence 2002 Chapman and Hall/CRC Press, Boca Raton, FL

